# USING SIMULATION TO ASSESS THE FIDELITY OF ADVANCE CARE PLANNING IN THE CONTEXT OF A PRAGMATIC TRIAL

**DOI:** 10.1093/geroni/igad104.1527

**Published:** 2023-12-21

**Authors:** Valerie Cotter, Danetta Sloan, Daniel Scerpella, Jennifer Wolff, Kelly Smith

**Affiliations:** Johns Hopkins University, Baltimore, Maryland, United States; Johns Hopkins Bloomberg School of Public Health, Baltimore, Maryland, United States; Johns Hopkins Bloomberg School of Public Health, Baltimore, Maryland, United States; Johns Hopkins University, Baltimore, Maryland, United States; University of Toronto, Toronto, Ontario, Canada

## Abstract

There is growing recognition of the importance of and challenges to maintaining fidelity in pragmatic randomized clinical trials. Simulations using standardized patients are a high-fidelity, low-stake, non-threatening opportunity to evaluate knowledge, skills, and competencies associated with high-quality healthcare delivery. We created standardized patient scenarios grounded in the Respecting Choices First Steps™ Advance Care Planning (ACP) curriculum to assess embedded trial ACP facilitators. Scenarios included simulations representing one-on-one encounters with a patient, and with a patient-family dyad. A standardized encounter observation checklist was used to assess and score relevant skills and behaviors including encounter set-up, ACP topics, and general communication. Each item was scored on a scale from not-done (0) to effective (2) with lower scores indicating lower fidelity. Six facilitators with varied backgrounds (social work, nursing, lay persons) each completed the two simulation scenarios. Group average domain scores across all six facilitators were moderately high. ACP Setup scoring averaged 75.5%; ACP Topics were 72.0%; and Communication were 77.4%. The lowest group scoring was observed in the coverage of ACP Topics (72%). The highest group average was observed in Communication skills at 84.9%. Lower individual scores were observed across all domains for staff who were newly hired at the time of the simulation exercise. Simulation using standardized patients and caregivers allowed investigators to monitor the fidelity of ACP communication to the trial design and provided targeted opportunities for improvement that were not readily available through usual care.

